# Nitroethanol in Comparison with Monensin Exhibits Greater Feed Efficiency Through Inhibiting Rumen Methanogenesis More Efficiently and Persistently in Feedlotting Lambs

**DOI:** 10.3390/ani9100784

**Published:** 2019-10-11

**Authors:** Zhen-Wei Zhang, Yan-Lu Wang, Yong-Yan Chen, Wei-Kang Wang, Luo-Tong Zhang, Hai-Ling Luo, Hong-Jian Yang

**Affiliations:** State Key Laboratory of Animal Nutrition, College of Animal Science and Technology, China Agricultural University, Beijing 100193, China; qingyibushuo@163.com (Z.-W.Z.); yanluwang@yeah.net (Y.-L.W.); yanz00@foxmail.com (Y.-Y.C.); 18292092306@163.com (W.-K.W.); zhangluotong@agri.gov.cn (L.-T.Z.); luohailing@cau.edu.cn (H.-L.L.)

**Keywords:** nitroethanol, monensin, growth performance, ruminal fermentation, methane emissions, feedlotting lambs

## Abstract

**Simple Summary:**

This study aimed to determine dietary supplemental effects of nitroethanol (NEOH) in comparison with monensin on growth performance, nutrient digestibility, rumen fermentation characteristics and methane production in feedlotting lambs. The dietary addition of NEOH in comparison with monensin presented a greater promoting effect on growth performance in feedlotting lambs by inhibiting methanogenesis more efficiently and persistently in the rumen. Although dietary NEOH or monensin addition did not affect nutrient digestibility in the whole digestion tract, they presented a distinct action mode of regulating ruminal volatile fatty acids (VFAs) and methane production.

**Abstract:**

This study was conducted to determine the dietary supplemental effects of nitroethanol (NEOH) in comparison with monensin on growth performance and estimated methane (CH_4_) production in feedlotting lambs. Sixty male, small-tailed Chinese Han lambs were arranged at random into three dietary treatment groups: (1) a basal control diet (CTR), (2) the basal diet added with 40 mg/kg monensin (MON), (3) the basal diet added with 277 mg/kg nitroethanol (NEOH). During the 32-day lamb feeding, monensin and nitroethanol were added in period 1 (day 0–16) and then withdrawn in the subsequent period 2 (day 17–32) to determine their withdrawal effects. The average daily gain (ADG) and feed conversion rate in the whole period ranked: NEOH > MON > CTR (*p* < 0.01), suggesting that the dietary addition of NEOH in comparison with monensin presented a more lasting beneficial effect on feed efficiency. Methane emission was estimated with rumen VFA production and gross energy intake. Both monensin and NEOH addition in comparison with the control remarkably decreased CH_4_ emission estimate (24.0% vs. 26.4% decrease; *p* < 0.01) as well as CH_4_ emission per kg ADG (8.7% vs. 14.0% decrease; *p* < 0.01), but the NEOH group presented obvious lasting methanogenesis inhibition when they were withdrawn in period 2. Moreover, the in vitro methanogenic activity of rumen fluids was also decreased with monensin or NEOH addition (12.7% vs. 30.5% decrease; *p* < 0.01). In summary, the dietary addition of NEOH in comparison with monensin presented a greater promoting effect on growth performance in feedlotting lambs by inhibiting rumen methanogenesis more efficiently and persistently.

## 1. Introduction

Methane (CH_4_), a potent greenhouse gas, originating from ruminant livestock is a growing threat to global warming [1,2], and it also causes an energetic loss of up to 12% of gross energy intake for the host animal [3]. Previous studies noted that some nitro compounds such as nitroethane, 2-nitroethanol, 2-nitro-1-propanol and 3-nitro-1-propionic acid were capable of inhibiting ruminal CH_4_-production in vitro [4,5,6,7,8]. Among these nitro compounds, nitroethane and 2-nitro-1-propanol were confirmed in vivo for their anti-methanogenic activity [9,10]. The earlier study by Anderson et al. [5] and a recent study by Zhang et al. [11] reported that nitroethane and 2-nitroethanol were nearly equally effective in inhibiting ruminal CH_4_ production in vitro, however, it is not clear if such methanogenesis inhibition could improve feed efficiency in growing farm animals.

Monensin, a polyether ionophore antibiotic, is well accepted as a routine feed additive to improve energy utilization and manipulate rumen fermentation. The benefits of feeding monensin to ruminant animals include increased milk yield [12], improved feed digestibility [13], enhanced energy metabolism and increased live weight gain [14,15,16]. The promoting action mode of monensin to improve energy efficiency is due to the fact that it can selectively inhibit gram-positive bacteria and shift rumen fermentation toward more propionate production and less CH_4_ emission [16,17].

As CH_4_ mitigation has the potential to improve feed energy efficiency, livestock producers may be more willing to adopt CH_4_ mitigation strategy in feeding practices. Until now, monensin has been included in ruminant rations to reduce enteric CH_4_ emission and improve feed conversion [18]. However, limited data is available concerning the effectiveness of nitroethanol (NEOH) in comparison with monensin to improve feed efficiency through inhibiting rumen methanogenesis. The present study was conducted to evaluate the dietary addition effect of NEOH in comparison with monensin and their withdrawal effect on ruminal fermentation, nutrient digestibility, growth performance and methanogenesis of fattening lambs.

## 2. Materials and Methods

In the present study, all of the procedures performed in animal feeding and sample collection followed the Guidelines of the Beijing Municipal Council on Animal Care (with protocol CAU20171014-1).

### 2.1. Chemicals

The light-yellow liquid nitroethanol product was purchased commercially from Sigma Aldrich (St. Louis, MO, USA) and stored at 4 °C, and its analytical grade was 90%. The ionophore sodium monensin was purchased from Beijing Lingrui Biotechnology Co., Ltd. (Beijing, China).

### 2.2. Animals, Diet Treatment and Sample Collection

Sixty male, small-tailed Chinese Han lambs (29.6 ± 0.41 kg body weight) were housed with four animals to a pen (2 m × 5 m) with bamboo slatted floors. All lambs were vaccinated for common infectious diseases and dewormed prior to the experiment. Pens of lambs were randomly assigned to one of three dietary treatments with five pens per treatment: (1) a basal control diet (CTR), (2) the basal diet added with 40 mg/kg sodium monensin (MON), (3) the basal diet added with 277 mg/kg 2-nitroethanol (NEOH) on dry matter basis (DM). The dosage level of monensin and 2-nitroethanol was referred to Soltan et al. [19] and Anderson et al. [5]. All the diets were prepared and fed as total mixed rations (TMR) and formulated to satisfy the nutrient requirements of growing lambs (Table 1). The lambs had free access to drinking water and were fed half the amount of TMR at 08:00 and the rest at 17:00. Regular adjustment of feed offered was made to avoid refusal amounts exceeding 10% in total ration offered. The whole feeding experiment consisted of period 1 (day 1–16) and a subsequent period 2 (day 17–32). In period 2, sodium monensin and 2-nitroethanol product were withdrawn, all three groups of lambs returned to be fed the same controlled diet.

During the whole experiment, daily feed offered and refusal were recorded at per pen level, and samples of each part were collected to determine DM content. Dry matter intake (DMI) was calculated as the difference between feed DM offered and DM refusal. Lambs were weighed at the start and end of period 1 and period 2 before morning feeding. Average daily gain (ADG) was calculated as the difference between the initial and final live body weight divided by the total days of feeding in each phase. Feed conversion rate (FCR) was calculated as ADG divided by DMI.

During the last three days of each period, feces from all lambs were collected by grab sampling through rectal palpation. Fecal samples were oven-dried at 65 °C over a four-day period and pooled within lamb. Dried feces were then ground to pass through a 1 mm screen and pooled equally within pen for chemical analysis. Rumen fluids (50 mL) were collected via esophagus 2 h after the morning feeding using an oral stomach tube (1.2 m length, 6.0 mm) connected to an adjustable vacuum pump. After discarding the initial 20 mL sample to eliminate saliva contamination, the remaining representative rumen fluids were strained through four layers of cheesecloth and sampled for the measurement of pH, ammonia N [20], microbial crude protein (MCP, [21]) and volatile fatty acids (VFAs, [22]). The remaining rumen fluids of four lambs per pen were mixed together equally and allocated into separate 100 mL glass bottles which were immediately capped to avoid exposure to air and then returned to the laboratory for immediate measurement of in vitro CH_4_ producing activity.

### 2.3. Analytics of CH_4_ Producing Activity

The combined rumen fluids (5 mL) of four lambs per pen were distributed into 120 mL glass bottles containing 10 mL freshly prepared buffer solution (pH 6.85; [23]) and 200 mg Chinese wildrye grass hay (ground to pass through a 1.0 mm screen). The bottles were incubated at 39 °C under a 100% N_2_ filled headspace. Cumulative gas production was measured at 3, 6, 12, 24, 36 and 48 h using the pressure transducer technique [24]. A three-way valve was used to collect the vented gas by connecting to pre-emptied gasbags. A 1.0 mL gas sample from the gasbags was injected into a gas chromatography for determination of CH_4_ concentrations [22].

### 2.4. Chemical Analyses

Representative samples of TMR offered and residues left were dried in a forced-air oven for the determination of initial moisture content. Samples of dried TMR, refusal and fecal were ground to pass through a 1 mm screen and analyzed following the Association of Official Analytical Chemists (AOAC; [25]) for dry matter (DM, ID 973.18), ash (ID 923.03), crude protein (CP, ID 4.2.08) or ether extract (EE, ID 920.85). Following the method of Van Soest et al. [26], neutral detergent fibre (aNDF) were determined with heat stable α-amylase and sodium sulphite addition and expressed inclusive of residual ash, and acid detergent fibre (ADF) were determined and expressed inclusive of residual ash. Acid insoluble ash (AIA) was determined following the method of Van Keulen and Young [27]. Briefly, ashed sample residues (550 °C) were boiled in 100 mL 4 N hydrochloric acid for 30 min, and subsequently filtered and washed free of acid with hot distilled water. Then, the ash and filter paper were transferred into a pre-weighed crucible and ashed 24 h at 550 °C in muffle stove again to determine the AIA content. In addition, the gross energy of TMR was measured by using the oxygen bomb calorimeter (MTZW–4, Shanghai Mitong Electromechanical Technology Co., Ltd., Shanghai, China). Following the method of Verdouw et al. [20], ammonia N in rumen fluid was measured at 637 nm wavelength colorimetrically using a microplate reader (RT-6500, Rayto Instruments, Shenzhen, China). Concentrations of MCP were determined based on the method of Makkar et al. [21] using Coomassie brilliant blue G-250 coloration solution (Solarbio Science & Technology Co., Ltd., Beijing, China) under a wavelength of 595 nm by the microplate reader. The concentrations of acetate, propionate, isobutyrate, butyrate, isovalerate and valerate were analyzed by a gas chromatography (GC522, Wufeng Instruments, Shanghai, China) equipped with a 15 m semicapillary column (Ø 0.53 mm) packed with Chromosorb 101, with pure N_2_ as the carrier gas at a column temperature of 120 °C [22]. For the determination of CH_4_ concentration, a 1 mL gas sample was injected to a gas chromatography packed with carbon porous beads (TDX-1) in a 2 m stainless steel column (2.0 mm inner diameter). The peaks of CH_4_ were identified by comparison with a standard of known composition [22].

### 2.5. Calculation

Ruminal CH_4_ production was estimated stoichiometrically based on the ruminal VFA concentrations according the prediction model of Moss et al. [28] as Equation (1):

CH_4_ (mmol/L) = 0.45 × acetate − 0.275 × propionate + 0.40 × butyrate (1)

Following the prediction model of Patra et al. [29], CH_4_ production (L/d) was calculated as follow based on the gross energy intake (GEI, MJ/d):

GEI (MJ/d) = DMI × GE(2)

CH_4_ (MJ/d) = 0.208 + 0.049 × GEI (3)

CH_4_ (L/d) = 0.714 × CH_4_ (MJ/d)/0.05565 (4)

GEI was calculated as Equation (2). The CH_4_ production in the present study was expressed as L/d, and the conversion of MJ/d to L/d was followed as Equation (4). When Equation (3) used MJ/d, a conversion factor (55.65 kJ per g of CH_4_) was used, and then the equation was reported in g/d, it was converted to L/d using the molar density of CH_4_ (0.714 g/L) [30].

In addition, measurements of CH_4_ production (L/kg ADG) were calculated in relation to the average daily gain.

Apparent total tract digestibility was calculated as Equation (5):

Nutrient digestibility (%) = 100 − 100 × (*N_F_* × *T_AIA_*)/(*N_T_* × *F_AIA_*) (5)

where *N_F_* is nutrient concentration in feces, *T_AIA_* is acid insoluble ash content in TMR, *N_T_* is nutrient concentration in TMR, *F_AIA_* is acid insoluble ash content in feces.

### 2.6. Statistical Analysis

Data were analyzed with diet type (Control, MON and NEOH), period (period 1 and period 2) and their interaction (diet × period) as the experimental factors having fixed effects using the MIXED procedure of SAS (SAS Inst. Inc., Cary, NC, USA; version 9.4) for a completely randomized design in two-way ANOVA according to the statistical model as Equation (6):

*Y_ijk_* = *μ* + *D_i_* + *P_j_* + (*D* × *P*)*_ij_* + *A_k_* + *e_ijk_* (6)

where *Y_ijk_* is dependent variables, *μ* is the overall mean, *D_i_* is the fixed effect of diet type (*i* = 3), *P_j_* is the fixed effect of period (*j* = 2, period 1 and period 2), (*D* × *P*) is the fixed effect of interaction between diet and period, *A_k_* is the random effect of animals (*k* = 60 per treatment) or pen (*k* = 5 per treatment), and *e_ijk_* is the random residual error. Least square means and standard errors of means were calculated with the LSMEANS procedure of the SAS software. Significance was declared at a level of *p* < 0.05 and trend at *p* ≤ 0.10.

## 3. Results

### 3.1. Effect of Monensin and NEOH on Nutrient Digestibility

The apparent digestibilities of DM, Organic matter (OM), CP, NDF and ADF did not differ among three groups in period 1, and almost all of them were decreased in the subsequent period 2. Although no significant difference among the diet treatments was observed during period 1 for the digestibility of ADF, it was decreased in NEOH group during the withdrawal period 2 (Table 2, *p* = 0.03). The interaction did not occur between diet and period for fermentation gas composition (*p* < 0.01).

### 3.2. Effect of Monensin and NEOH on Feed Intake and Growth Performance

**Figure 1:**
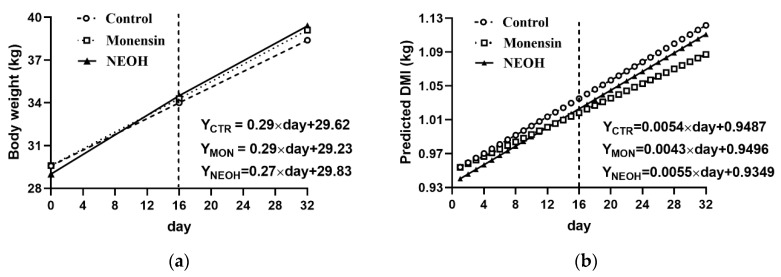
Supplementation effects of monensin (MON) and nitroethanol (NEOH) to lamb diet in feeding period 1 (day 0–16) and their withdrawn effects in subsequent period 2 (day 17–32) on live body weight (**a**) and dry matter intake (DMI, **b**).

The final body weight (BW) of fattening lambs did not differ among the three treatment groups (Table 3 and Figure 1a, *p* >0.05). The dietary addition of monensin or NEOH tends to decrease DMI (Table 3 and Figure 1b, *p* = 0.08). Both MON and NEOH groups increased ADG and FCR (Table 3, *p* < 0.01). The ADG in both period 1 and period 2 ranked: NEOH > MON > CTR (*p* < 0.01). Interaction effects occurred between diet × period for FCR (*p* < 0.01). The FCR in period 1 ranked: NEOH > MON > CTR (*p* < 0.01), and dietary addition of NEOH or MON nearly equally increased FCR in period 2.

### 3.3. Effect of Monensin and NEOH on Ruminal Fermentation

Interaction effects (Table 4, *p* < 0.05) occurred between diet × period for pH, total VFA, acetate, propionate and butyrate. In addition, the period also affected (*p* < 0.01) these fermentation characteristics (*p* < 0.05). The dietary addition of monensin or NEOH increased rumen pH (*p* < 0.01), and the pH increment in period 2 against period 1 showed a greater lasting effect in NEOH than the MON group. The NEOH addition decreased ammonia N (*p* < 0.01) and tended to increase MCP (*p* = 0.09) in the rumen, and such effects were not observed in the MON addition group. Dietary addition of MON decreased total VFA in the rumen (*p* < 0.01), but such an effect did not occur in the NEOH group in both periods 1 and 2. 

Regarding the VFA pattern in molar percentage, compared with the control both MON and NEOH group decreased butyrate (22.3% vs. 15.8% decrease; *p* < 0.01). Dietary monensin addition increased propionate by 11.4% while dietary NEOH addition increased acetate by 5.0% (*p* < 0.01). Consequently, the acetate to propionate ratio (A:P) in period 1 was decreased in MON group and increased in NEOH group, but the A:P ratio shift as well as the butyrate decrease disappeared in period 2.

### 3.4. Effect of Monensin and NEOH on CH_4_ Emissions and In Vitro CH_4_ Producing Activity

Methane emission was estimated with the models of Moss et al. [28] based on rumen VFA production and Patra et al. [29] based on GEI (Table 5). Interaction effects (*p* < 0.05) occurred between diet × period for CH_4_ emission. Both MON and NEOH addition in comparison with the control decreased CH_4_ emission (24.0% vs. 26.4% decrease; *p* < 0.01) as well as methane emission per kg ADG (8.7% vs. 14.0% decrease; *p* < 0.01), but the latter NEOH group presented obvious lasting methanogenesis inhibition when they were withdrawn in period 2. In vitro CH_4_ producing activity was decreased with either monensin or NEOH addition (12.7% vs. 30.5% decrease; *p* < 0.01). In addition, the NEOH in comparison with MON exhibits greater anti-methanogenic activity in both periods 1 and period 2 (*p* < 0.01).

## 4. Discussion

Regarding the negative issues of enteric CH_4_ production from ruminants [3,31], considerable efforts have evolved for mitigating CH_4_ emission. Until now, monensin has been commonly used in feeding practice of ruminant diets to decrease CH_4_ yield and improve feed efficiency. The anti-methanogenic potential of monensin has been demonstrated both in vitro and in vivo studies [19,31,32]. However, an efficient effect of nitroethanol on the reduction of CH_4_ production (>90%) has recently been reported in vitro [5,6,7], the in vivo results investigating potential effects of nitroethanol on CH_4_ mitigation are lacking in comparison with monensin addition. Additionally, to our knowledge, research regarding the practical use of nitro compounds and thereafter the effect of nitro compounds addition on animal productivity, growth performance and nutrient digestibility has not been reported until now.

### 4.1. Effect of NEOH in Comparison with Monensin on Feed Intake, Growth Performance and Diet Digestibility

Due to the antimicrobial properties and its efficacy in promoting propionate synthesis, the dietary addition of monensin often resulted in a depression of DMI [33,34]. The increased propionate production would supply additional energy for ruminant and consequently decrease feed intake through inhibiting the activity of the feeding centre of hypothalamus. In addition, it has been noted that dietary monensin addition decreased rumen motility and influenced the dilution rate of digestion of nutrients [35]. This results in an increase of ruminal fill and a reduction of DMI [36], and this could explain why daily DMI in the present study tended to decrease in both period 1 and period 2 with monensin supplementation. Nevertheless, the effects of monensin on DMI could also depend on various other factors including dietary composition, the dose level of monensin supplemented and mode of delivery [13]. Both Chapman et al. [37] and Monnerat et al. [38] reported no effect of monensin on DMI in ruminants. Moreover, to the best of our knowledge, the current study is the first to report showing a similar negative effect of NEOH on DMI in feedlotting lambs. 

The positive impacts of monensin as dietary antibiotic growth promoters on livestock productivity have been well-documented [37,39]. Presently, the ADG and feed conversion was improved with monensin addition during both periods 1 and 2. Except for the relative low feed intake, the greater propionate yield and lower enteric CH_4_ emissions in response to monensin could be an explanation for the improvement of ADG and feed conversion. In the rumen, gram-positive bacteria are responsible for producing H_2_, CH_4_, ammonia and lactate [16], while gram-negative bacteria are considered propionic acid producers [40]. Due to the antimicrobial properties of gram-positive bacteria, it is well known that monensin has an ability to shift the ruminal bacterial communities from gram-positive to gram-negative organisms [41]. Therefore, the selective inhibition of gram-positive bacteria and enteric CH_4_ reduction occurred in the present study with monensin addition, and, ultimately, promoted the ADG and feed efficiency in feedlotting lambs. Meanwhile, the NEOH addition also increased the ADG and feed efficiency, however, the molar proportion of propionate was not affected by the NEOH addition. In contrast, the molar proportion of acetate was increased with NEOH supplementation. Thus, the action mode of monensin and NEOH on improving energy efficiency was different. Moreover, the current study is the first research to demonstrate the positive effect of NEOH on animal growth performance and it needs further intensive studies in the future. 

During the period 1 with monensin and NEOH supplementation, apparent digestibility of nutrients was not affected by both NEOH and monensin. In agreement with the current study, previous studies have reported that apparent total tract digestibility of nutrients was not altered with monensin supplementation in lactating cows, feedlot heifers, or growing lambs [32,42,43]. According to Benchaar et al. [34] and Plaizier et al. [44], however, the supplementation of different doses of monensin in dairy cows increased the CP digestibility. The improved CP digestibility could be attributed to the inhibitory effect of monensin on ruminal hyper-ammonia-producing and obligate-ammonia-degrading microbes which could increase the fraction of dietary protein escaping the rumen and increasing its post ruminal availability [32,45]. However, withdrawn of monensin and NEOH supplementation during period 2, the apparent digestibility of CP was lower in monensin-fed lambs. Thus, the antimicrobial effects of monensin on hyper-ammonia-producing and obligate-ammonia-degrading microbes might be diminished during withdrawal period 2 without the addition of monensin. Until now, the effect of NEOH on apparent digestibility of nutrient was first determined in the present study, and results showed that NEOH had no negative effect on the apparent digestibility of nutrient. Although the more thoroughly and intensive pieces of evidence remain to be explored, the present results indicated that NEOH could be a potent CH_4_ inhibitor that can be added to conventional feedlot diets without incurring adverse effects on the apparent digestibility of nutrient.

### 4.2. Effect of Monensin and NEOH on Ruminal Fermentation Profiles

As an intermediate product, ammonia N content reflects a balance between its release from dietary protein degradation and its uptake by rumen microorganism to synthesize MCP [19]. In accordance with previous work by Cochran et al. [46] and Fredrickson et al. [47], the supplementation of monensin had no effect on ruminal ammonia N and MCP concentration. However, some other studies have previously been observed to reduce ruminal ammonia N concentration by monensin [48], suggesting an inhibitory effect of monensin on dietary protein degradation. The differences among these results could be explained by the fact that protein degradation in the rumen not only involved the deamination of dietary protein but also included proteolysis and peptidolysis processes. In contrast, the supplementation of NEOH in the present study decreased the ruminal ammonia N concentrations while enhanced the MCP concentrations, suggesting a promotion effect on N utilization efficiency by rumen microbes. 

During period 1 with monensin and NEOH supplementation, NEOH decreased the total VFA concentrations while monensin had no significant effect on total VFA productions. Limited in vivo data is available concerning the effect of NEOH on total VFA concentrations. In some earlier in vitro results [6,9], NEOH was observed to have no negative effect on total VFA. However, the effects of NEOH on total VFA production might depend on the dose and duration of NEOH supplementation, as well as dietary composition. In addition, ruminal VFAs were either the catabolism-products of dietary degradation or important energy source that be utilized by the host. The effects of monensin supplementation on total VFA concentration varied among previous studies [49,50,51], which may be attributed to the duration of monensin treatment. In period 2 without monensin and NEOH supplementation, ruminal VFA concentrations were increased significantly in both monensin and NEOH group when compared to period 1. Taken together, the present result suggested that NEOH in comparison with monensin in the previous study [52] did not present an inhibitory effect on total VFA. 

Ruminal propionate is an important substrate for hepatic gluconeogenesis, and its increase indirectly reflects a promotion of glucose synthesis for host ruminants. Moreover, propionate production during rumen fermentation is always accompanied by H_2_ consumption [53]. In agreement with previous findings [54,55], monensin remarkably increased the molar proportions of propionate in the current study. Russell et al. [56] noted that monensin had a selective inhibition effect on ruminal microbes, resulting in a decrease of the acetate-to-propionate ratio by diverting reducing equivalents towards propionate synthesis in the rumen. However, with the adaptation of several members of gram-positive bacteria through modifying cell wall structure or development of resistance, monensin might lose its specific effect (e.g., propionate increase) as time progresses. Therefore, the propionate proportions declined during withdrawal period 2 without monensin in comparison with period 1. Unlike monensin, NEOH altered the VFA pattern towards the production of acetate acid rather than that of propionate acid. Acetate, however, is nonglucogenic; rather, it is a precursor for long-chain fatty acid synthesis. As a result, ruminal VFA pattern of the present assay showed the ability of NEOH to modify the rumen fermentation differently to what occurred in the monensin treatment. In addition, the current results agreed with results from in vitro studies that the propionate production was unaffected by nitro compounds including nitroethane, nitroethanol and nitropropanol [5,6,57].

### 4.3. Effect of Monensin and NEOH on CH_4_ Emissions

The ruminal production of acetate or butyrate is often accompanied by H_2_ production, whereas propionate formation is associated with H_2_ consumption [28]. Therefore, promoting propionate production is one of the optimal ruminal pathways to reduce CH_4_ production from ruminant animals [58]. Monensin reduced the acetate-to-propionate ratio by diverting H_2_ availability towards propionate acid synthesis [59,60], and this could explain partially the inhibition to CH_4_ production in the present study. Methane emission was estimated with the models of Moss et al. [28] based on rumen VFA production and Patra et al. [29] based on GEI. Both monensin and NEOH addition in comparison with the control reduced CH4 emissions. However, in contrast with the study of Li et al. [52] who have reported 20.3% reduction in CH_4_ emission in goats by monensin, the enteric CH_4_ production was not reduced by monensin and NEOH when expressed as L/day. This was consistent with the findings of Hemphill et al. [44] and Guan et al. [61] in heifers and steers. Due to the principal interest of the livestock producers to redirect CH_4_ reduction towards the promotion of live weight gain [62], CH_4_ emission relative to the unit of host product (e.g., L/kg ADG) is more important than the animal’s daily production (e.g., L/day) [19]. The feedlotting lambs treated with both monensin and NEOH had the lower predicted CH_4_ emission values expressed relative to the ADG (L/kg ADG; [28]) when they were compared with the control. According to Johnson and Johnson [3] and Guan [61], the reduction in CH_4_ emission by monensin may not persist over time. In withdrawal period 2 without monensin addition, CH_4_ emissions predicted by the model of Moss et al. [28] recovered to the same level of the control. However, both monensin and NEOH in the present study still exhibited significant inhibition of CH_4_ production expressed relative to the ADG in period 2.

Numerous in vitro studies have documented the anti-methanogenic activity of nitrocompounds including nitroethane, NEOH and nitropropanol [5,6,7,8]. In addition, results from in vivo studies provided further evidence that the nitro compounds such as nitroethane and nitropropanol was able to inhibit CH_4_ production in ovine and bovine [9,10]. However, the present study is the first in vivo study showing the ability of NEOH to reduce CH_4_ emissions in feedlotting lambs. Although the inhibitory mechanism of action of NEOH remains unclear, it is different from the monensin mechanism. In the present study, NEOH inhibited ruminal methanogenesis without adversely affecting the ratio of acetate to propionate. A review reported by Zhang et al. has shown that nitro compounds possibly by inhibiting H_2_ and formate oxidation, serving as electron acceptors within rumen microbial populations or exerting a direct inhibition of ruminal methanogens to inhibit in vitro CH4 production [6]. However, more thorough knowledge of the rumen microbial population is needed to better understand the NEOH action model of antimethanogenic activity. Additionally, the use of dietary additive raises food safety and public concerns with respect to livestock product and animal health. Fortunately, no apparent symptoms of toxicity were observed during the whole experimental period for both NEOH- and monensin-treated lambs. 

## 5. Conclusions

The dietary addition of NEOH in comparison with monensin presented a greater promoting effect on growth performance in feedlotting lambs by inhibiting methanogenesis more efficiently and persistently in the rumen. Although dietary NEOH or monensin addition did not affect nutrient digestibility in the whole digestion tract, they have a distinct mode of action regulating microbial VFAs and CH_4_ production in the rumen. In addition, we conclude that NEOH is a potent CH_4_ inhibitor that could be added to conventional feedlot diets without incurring negative effects on digestibility and performance.

## Figures and Tables

**Table 1:** Ingredients and nutrient level of the basal diet for growing lambs.

Item	Basal Diet
Ingredients (g/kg, as fed basis)	
Corn silage	600
Peanut vine	100
Corn meal	109.5
Wheat bran	30
Soybean meal	150
Limestone	3
Sodium bicarbonate	1.5
Salt	3
Premix ^1^	3
Nutrient level (g/kg, as Dry Matter) ^2^	
Organic matter	944
Crude protein	163
Ether extract	22
Neutral detergent fiber	360
Acid detergent fiber	223
Calcium	5.5
Phosphorus	4.5
Gross energy (MJ/kg, as Dry Matter)	15.19

^1^ The mineral-vitamin premix provided nutrients per kg of diet: Mn, 64 mg; Fe, 56 mg; Zn, 45 mg; Cu, 9.6 mg; Se, 0.3 mg; I, 1 mg; vitamin A, 48,000 IU; vitamin D, 11,000 IU; vitamin E, 33 IU; folic acid, 1.0 mg; nicotinic acid, 60 mg; d-calpanate, 30 mg and d-biotin, 0.1 mg; ^2^ Determined using samples pooled by diet three times within each week.

**Table 2:** Supplementation effects of monensin and nitroethanol (NEOH) to lamb diet in feeding period 1 (day 0–16) and their withdrawn effects in subsequent period 2 (day 17–32) on total tract apparent digestibility.

Digestibility (g/kg)	Treatments			SEM	*p*–Value		
	Control	Monensin	NEOH		Diet	Period	Diet × Period
Dry matter							
Period 1	780 ^A^	768 ^A^	771 ^A^	8.0	0.08	<0.01	0.46
Period 2	716 ^B^	701 ^B^	688 ^B^				
Organic matter							
Period 1	800 ^A^	788 ^A^	792 ^A^	8.1	0.10	<0.01	0.43
Period 2	735 ^B^	723 ^B^	708 ^B^				
Crude protein							
Period 1	777 ^A^	767 ^A^	765 ^A^	8.1	0.02	<0.01	0.25
Period 2	714 ^Ba^	678 ^Bb^	696 ^Bab^				
Neutral detergent fibre							
Period 1	651 ^A^	632 ^A^	635 ^A^	17.1	0.14	<0.01	0.33
Period 2	583 ^B^	576 ^B^	529 ^B^				
Acid detergent fibre							
Period 1	647 ^A^	613 ^A^	629 ^A^	15.9	0.03	<0.01	0.08
Period 2	593 ^Ba^	575 ^Ba^	519 ^Bb^				

^a, b^ Means within a row with different lowercase superscript letter differ at *p* < 0.05; ^A, B^ Means within a column with different uppercase superscript letter differ at *p* < 0.05; SEM, standard error of the mean.

**Table 3:** Supplementation effects of monensin and nitroethanol (NEOH) to lamb diet in feeding period 1 (day 0–16) and their withdrawn effects in subsequent period 2 (day 17–32) on live body weight (BW), dry matter intake (DMI) and growth performance.

Item	Treatments			SEM	*p*–Value		
	Control	Monensin	NEOH		Diet	Period	Diet × Period
Initial BW (kg)	29.58	29.61	29.57	0.73	0.99	NA	NA
Final BW (kg)							
Period 1	34.0 ^B^	34.3 ^B^	34.5 ^B^	0.79	0.66	<0.01	0.88
Period 2	38.4 ^A^	39.1 ^A^	39.4 ^A^				
DMI (g/day)							
Period 1	1012 ^B^	1004 ^B^	996 ^B^	9.2	0.08	<0.01	0.13
Period 2	1100 ^A^	1065 ^A^	1093 ^A^				
ADG (g)							
Period 1	272 ^c^	293 ^Bb^	310 ^a^	2.1	<0.01	0.17	0.06
Period 2	273 ^b^	301 ^Aa^	309 ^a^				
FCR							
Period 1	0.27 ^Ac^	0.29 ^b^	0.31 ^Aa^	0.003	<0.01	<0.01	0.01
Period 2	0.25 ^Bb^	0.29 ^a^	0.29 ^Ba^				

^a, b, c^ Means within a row with different lowercase superscript letters differ at *p* < 0.05; NA, not applicable; ^A, B^ Means within a column with different uppercase superscript letters differ at *p* < 0.05; ADG, average daily gain; DMI, dray matter intake; FCR, feed conversion ration calculated as ADG divided by DMI; SEM, standard error of the mean.

**Table 4:** Supplementation effects of monensin and nitroethanol (NEOH) to lamb diet in feeding period 1 (day 0–16) and their withdrawn effects in subsequent period 2 (day 17–32) on rumen fermentation characteristics.

Item	Treatments			SEM	*p*–Value		
	Control	Monensin	NEOH		Diet	Period	Diet × Period
pH							
Period 1	6.05 ^Bb^	6.39 ^a^	6.26 ^Ba^	0.047	<0.01	<0.01	<0.01
Period 2	6.29 ^Ab^	6.26 ^b^	6.56 ^Aa^				
NH_3_N, g/L							
Period 1	35.5 ^a^	33.6 ^a^	20.9 ^Bb^	0.96	<0.01	0.03	0.09
Period 2	34.8 ^a^	34.8 ^a^	27.7 ^Ab^				
MCP, mg/mL							
Period 1	0.59 ^Ab^	0.60 ^Ab^	0.66 ^Aa^	0.024	0.09	<0.01	0.34
Period 2	0.43 ^B^	0.45 ^B^	0.44 ^B^				
Total VFA, mmol/L							
Period 1	121.6 ^a^	115.0 ^Ba^	100.4 ^Bb^	2.18	<0.01	<0.01	<0.01
Period 2	123.1 ^a^	125.1 ^Aa^	114.6 ^Ab^				
VFA patterns (% molar)							
Acetate							
Period 1	59.7 ^Bb^	60.4 ^b^	62.7 ^a^	0.56	<0.01	0.01	0.04
Period 2	62.5 ^A^	61.4	62.6				
Propionate							
Period 1	20.1 ^b^	22.4 ^Aa^	19.5 ^b^	0.48	<0.01	<0.01	0.01
Period 2	19.2	18.9 ^B^	18.4				
Butyrate							
Period 1	13.9 ^a^	10.8 ^Bb^	11.7 ^Bb^	0.45	0.04	<0.01	<0.01
Period 2	13.3	14.4 ^A^	13.3 ^A^				
BCVFA							
Period 1	5.1 ^A^	5.2 ^A^	5.1	0.17	0.22	<0.01	0.06
Period 2	4.5 ^B^	4.6 ^B^	4.7				
Acetate: Propionate ratio							
Period 1	3.0 ^a^	2.7 ^Bb^	3.3 ^a^	0.09	<0.01	<0.01	0.08
Period 2	3.3	3.3 ^A^	3.4				

^a, b^ Means within a row with different lowercase superscript letter differ at *p* < 0.05; ^A, B^ Means within a column with different uppercase superscript letter differ at *p* < 0.05; NH_3_N, ammonia N; MCP, microbial protein; VFA, volatile fatty acids; BCVFA, branch-chained volatile fatty acids including iso-butyrate and iso-valerate; SEM, standard error of the mean.

**Table 5:** Supplementation effects of monensin and nitroethanol (NEOH) to lamb diet in feeding period 1 (day 0–16) and their withdrawn effects in subsequent period 2 (day 17–32) on methane emission.

Item	Treatments			SEM	*p*–Value		
	Control	Monensin	NEOH		Diet	Period	Diet × Period
Methane, mmol/L ^1^							
Period 1	25.8 ^a^	19.6 ^Bb^	19.0 ^Bb^	0.59	<0.01	<0.01	<0.01
Period 2	26.9 ^a^	26.2 ^Aa^	24.2 ^Ab^				
Methane, L/day ^2^							
Period 1	12.3 ^B^	12.2 ^B^	12.2 ^B^	0.09	0.08	<0.01	0.13
Period 2	13.2 ^A^	12.8 ^A^	13.1 ^A^				
Methane, L/kg ADG ^2^							
Period 1	45.8 ^Ba^	42.0 ^b^	39.3 ^Bc^	0.44	<0.01	<0.01	0.01
Period 2	48.4 ^Aa^	42.6 ^b^	42.7 ^Ab^				
In vitro CH_4_ producing activity ^3^							
Period 1	11.8 ^Aa^	10.3 ^b^	8.2 ^Bc^	0.15	<0.01	<0.01	<0.01
Period 2	11.1 ^Ba^	10.1 ^b^	9.2 ^Ac^				

^a, b, c^ Means within a row with different lowercase superscript letter differ at *p* < 0.05; ^A, B^ Means within a column with different uppercase superscript letter differ at *p* < 0.05; ^1^ CH_4_ emission was estimated with the model of Moss et al. based on rumen VFA production.; ^2^ CH_4_ emission was estimated with the model Patra et al. based on DMI; ^3^ CH_4_-producing activity was measured by in vitro incubation of 5 mL ruminal fluid with 10 mL freshly prepared buffer solution and 200 mg Chinese wildrye grass hay. The vented gas was collected by pre-emptied gasbags for later analysis of CH_4_ content through gas chromatography; SEM, standard error of the mean.
